# An Authentication and Key Management Mechanism for Resource Constrained Devices in IEEE 802.11-based IoT Access Networks

**DOI:** 10.3390/s17102170

**Published:** 2017-09-21

**Authors:** Ki-Wook Kim, Youn-Hee Han, Sung-Gi Min

**Affiliations:** 1 Department of Computer and Radio Communication Engineering, Korea University, Seoul 136-713, Korea; wowook@gmail.com; 2 School of Computer Science and Engineering, Korea University of Technology and Education, CheonAn 330-708, Korea; yhhan@koreatech.ac.kr; 3 Department of Computer Science and Engineering, Korea University, Seoul 136-713, Korea

**Keywords:** Internet of Things (IoT), IEEE 802.11ah, resource constrained device, access network security, authentication and key management (AKM)

## Abstract

Many Internet of Things (IoT) services utilize an IoT access network to connect small devices with remote servers. They can share an access network with standard communication technology, such as IEEE 802.11ah. However, an authentication and key management (AKM) mechanism for resource constrained IoT devices using IEEE 802.11ah has not been proposed as yet. We therefore propose a new AKM mechanism for an IoT access network, which is based on IEEE 802.11 key management with the IEEE 802.1X authentication mechanism. The proposed AKM mechanism does not require any pre-configured security information between the access network domain and the IoT service domain. It considers the resource constraints of IoT devices, allowing IoT devices to delegate the burden of AKM processes to a powerful agent. The agent has sufficient power to support various authentication methods for the access point, and it performs cryptographic functions for the IoT devices. Performance analysis shows that the proposed mechanism greatly reduces computation costs, network costs, and memory usage of the resource-constrained IoT device as compared to the existing IEEE 802.11 Key Management with the IEEE 802.1X authentication mechanism.

## 1. Introduction

Currently, the rapid growth of the Internet of Things (IoT) is facilitating the investigation of various wireless communication technologies for small devices with low cost air interfaces. IoT devices use low-cost, low-speed communication technologies such as those presented in [1,2,3,4]. While these technologies enable local connections among IoT devices, they also need to be connected to the Internet for various purposes, such as data collection, remote control and device management. Standardizing access network technologies for Internet connectivity of IoT devices is continuing as well.

**Figure 1:**
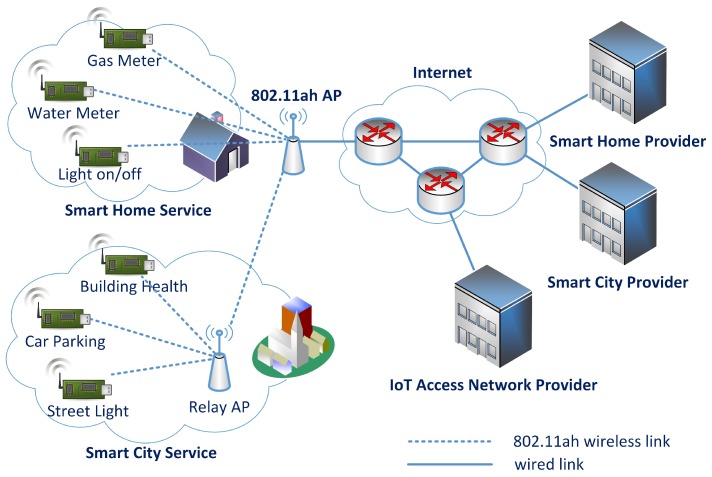
IEEE 802.11ah Network Model.

The IEEE 802.11ah Task Group (TGah) is working on a new standard to address the particular requirements of IoT access networks. It uses low frequency bands enabling low power consumption and a wider coverage range (1 km radius for an Access Point (AP)), and it can be extended by relay APs. Its coverage range is sufficient to connect all small devices for multiple IoT services in a building to an AP. Figure 1 shows a network model in which an IEEE 802.11ah AP provides network access to multiple IoT services. While the AP belongs to the domain of the IoT access network provider, the IoT devices belong to each IoT service provider domain. Heterogeneous IoT devices can share the access network for Internet connectivity.

Consideration must be given to the security between heterogeneous IoT devices and the AP. Initially, the access network domain and the IoT service domains do not trust each other. They need to confirm the service authority and establish a secure communication channel through an authentication and key management (AKM) mechanism. The AKM mechanism should support various negotiable authentication methods for various IoT services, while also considering the resource constraints of the IoT devices.

In this paper, we propose a new AKM mechanism for an IoT access network to establish a security association (SA) between a resource-constrained IoT device and an AP. The proposed mechanism is based on the IEEE 802.11 key management with the 802.1X authentication mechanism. It introduces a station-side authentication server (SAS). The IoT devices delegate most of the burden of authentication and key derivation to the SAS and only need to verify mutual authenticity with the AP by using basic encryption and decryption functions that devices already possess for data security. Another advantage of this delegation by IoT devices is that the security algorithms used for deriving the session key are independent of the IoT devices and thus can be replaced with other algorithms without affecting the IoT devices. The mechanism also reduces the authentication workload of the access network. The AP only authenticates the SAS once at the attachment of the first device belonging to the SAS, and the SAS can guarantee the authenticity of all subsequent IoT devices that are registered at the SAS.

## 2. IEEE 802.11 Authentication and Key Management Mechanism

In IEEE 802.11, IEEE 802.1X-2004 [5] is used as the default authentication mechanism, and the key management is described in IEEE 802.11-2012 [6], Section 11. IEEE 802.1X describes the Extensible Authentication Protocol [7] over LAN (EAPOL) mechanism that conveys the EAP message and session key materials over the LAN environment. The EAPOL-EAP is used for authentication and the EAPOL-KEY is then used for the key management. By using the EAPOL mechanism, IEEE 802.11 offloads the authentication responsibility from the authenticator (the AP) to the authentication server (AS). It is assumed that the AS and the AP have a SA and that the master shared key (MSK) sent from the AS to the AP will not be compromised on either side.

**Figure 2:**
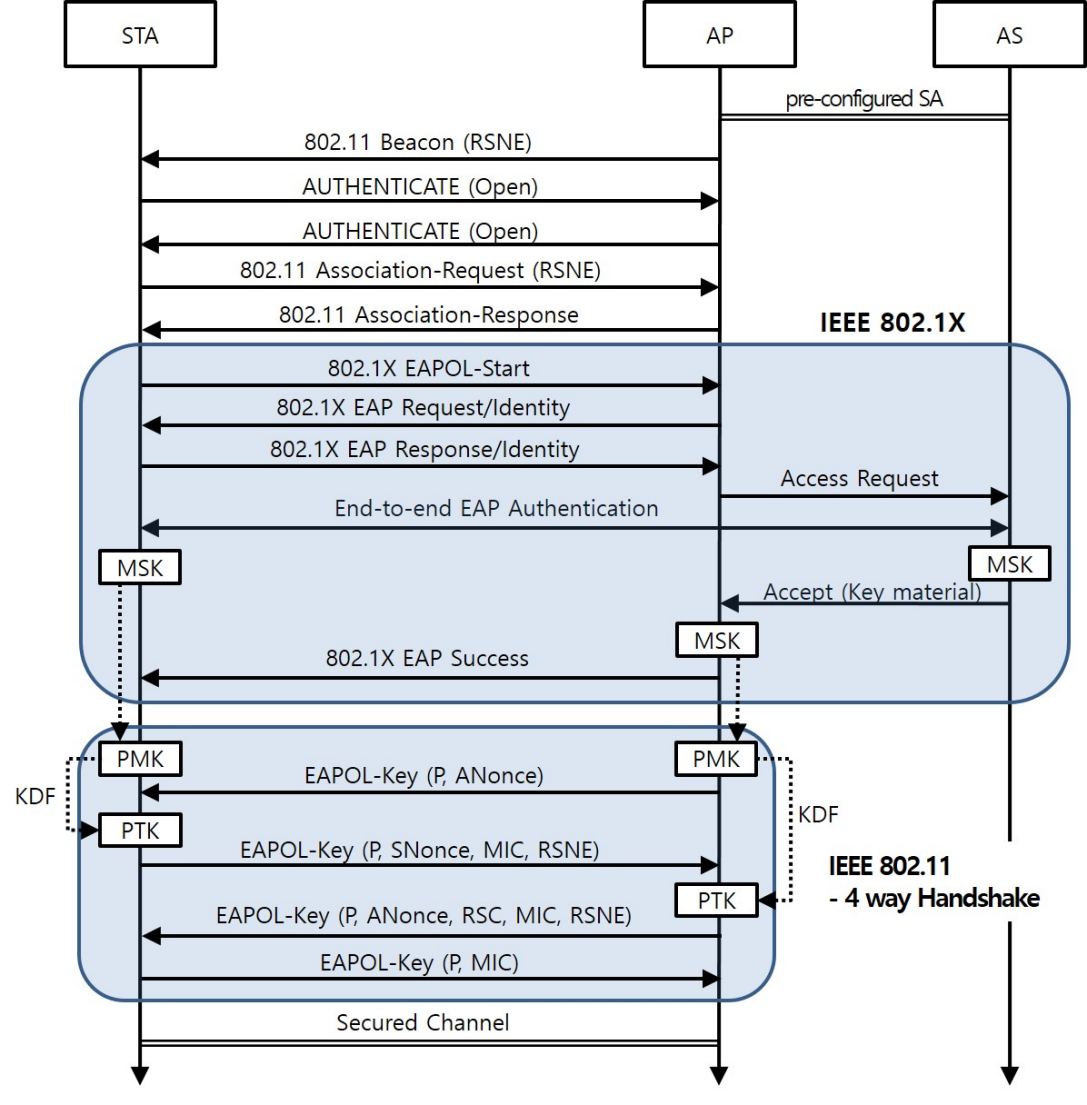
Sequence Diagram of the New Node Attachment in IEEE 802.11 with IEEE 802.1X.

Figure 2 presents a sequence diagram for the SA establishment between a station (STA) and the AP to which the STA is associated. Table 1 specifies the notations in the figure. The SA establishment is performed in two phases. Firstly, on behalf of the AP, a STA and the AS authenticate each other and generate a MSK. The AS notifies the AP of the results of the mutual authentication and delivers the MSK to the AP. Then, the session keys are derived from the MSK at the STA and the AP. The four-way handshake is used at this phase.

## 3. IoT Authentication Architecture

**Figure 3:**
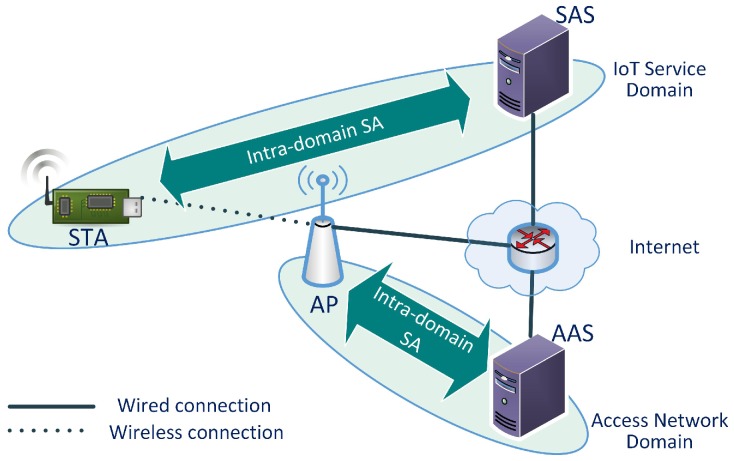
IoT Authentication Architecture.

Figure 3 shows the network architecture for the proposed mechanism. The network has two types of domains: the IoT service domain and the access network domain. An IoT service domain consists of STAs and a SAS. The access network domain consists of APs and the AP-side authentication server (AAS). Multiple IoT service domains can share the same access network. Each IoT service domain has a unique domain identifier. The intra-domain SA between the STA and the SAS in the figure indicates that there is a pre-configured Security Association between them. However, in order for them to communicate directly, the STA must access the internet. It is possible after the AKM has been successfully finished and the AP allows the STA to access the network.

### 3.1. STA

A station (STA) is a resource-constrained IoT device. It provides basic data security services (DSS), taken from the IEEE 802.15.4 security service [8], to exchange its sensitive data via an unsecured wireless medium. The DSS should include data confidentiality, data authenticity, and replay protection. We assume that the STA incorporates the minor variation of the AES-CCM, which is called AES-CCM* in IEEE 802.15.4, as its cryptographic mechanism within the DSS. The CCM mode combines the counter (CTR) mode for data confidentiality and the CBC-MAC (CMAC) mode for authentication and integrity. The CCM* mode has all the features of the CCM and offers encryption-only and integrity-only capabilities additionally. The proposed AKM protocol in this paper also serves a cryptographic function in the DSS, considering the memory resource constraints of the IoT device. Thus, the AES-CMAC is used for message integrity code (MIC) calculation and the AES-CTR is used for key data encryption and decryption (AES-CTR is adopted as a key data encryption algorithm of Protocol for carrying Authentication for Network Access (PANA) [9]). The EAPOL-KEY messages are then used to set the session key for the DSS to the STA, and they include Key Replay Protection as IEEE 802.11-2012. A STA connects with an AP in the access network domain and then starts the authentication phase. The STA delegates the supplicant role of authentication to the SAS. A STA has the SAS identifier, a SAS-registered Pre-Shared Key (PSK), and the list of access network identifiers to which it can connect.

### 3.2. SAS

A station-side authentication server (SAS) is the authentication server for STAs. A STA is registered at the SAS before deployment. During this registration, intra-domain SA information such as PSK will be printed on the device, and SAS will store the STA-ID and the PSK of the device. The PSK is used as the key-encryption key (KEK) between the SAS and the STA. The SAS guarantees the authenticity of the STAs registered to the AP of which the STAs are associated. The SAS also has the cryptographic functions used for key derivation, such as random number generators (RNG) and key derivation functions (KDF), on behalf of the STAs. The transport protocol for EAPOL messages between the AP and the SAS can use any layer 2 tunneling protocol, such as L2TP, etc. Each SAS has an identifier in the form of a Network Access Identifier [10]. The identifier is a string up to 100 octets long. STAs need to know the identifier of the SAS at which they registered.

### 3.3. AP

An access point (AP) is an access network entity that provides a point of attachment to STAs. It has an access network identifier pointing to the access network to which it belongs. In the access network domain, the AP enforces access control for the STAs that are attached to the access network. The AP uses the IEEE 802.11ah-2016 authentication control mechanism to control the number of simultaneously associating STAs. The AP then establishes a SA with a STA and acts as the authenticator for the STA. The AP must be able to identify the SAS by the SAS identifier.

### 3.4. AAS

The AP-side authentication server (AAS) acts as an authentication server in IEEE 802.1X. The AAS guarantees the authenticity of the registered APs to the SAS. An AP and the AAS have a pre-configured SA. The EAP messages between the AP and the AAS can use any Authentication, Authorization, and Accounting (AAA) transport protocol. In this paper, we use the RADIUS.

## 4. Proposed Authentication and Key Management Procedure

**Figure 4:**
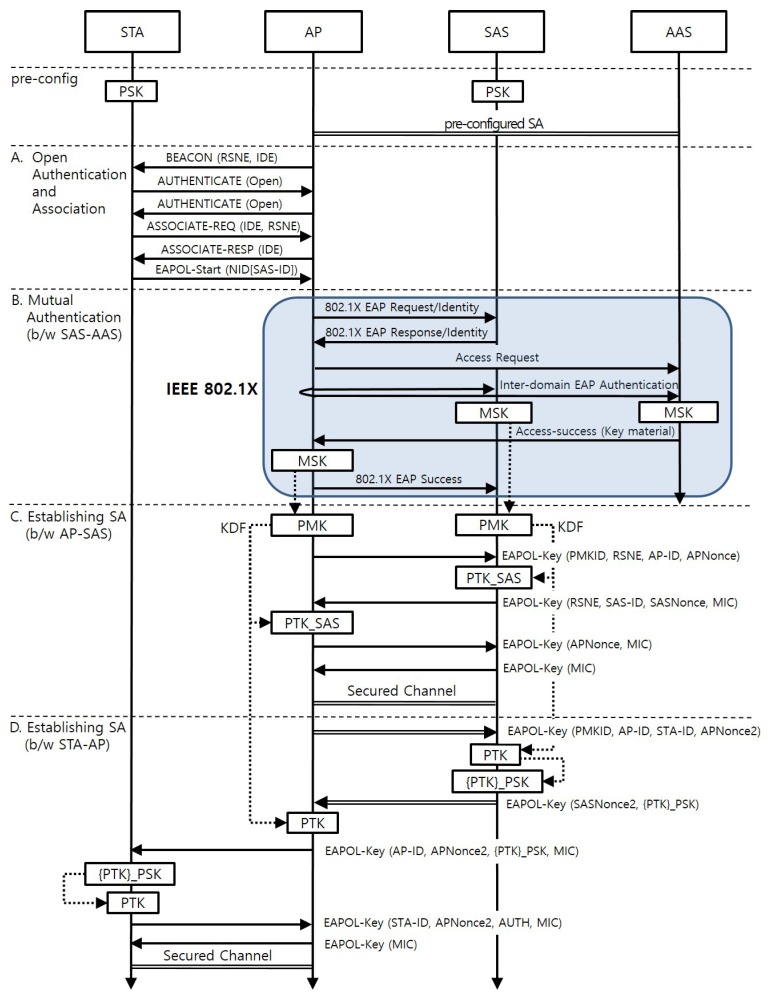
Sequence Diagram of the Proposed Protocol.

Figure 4 shows the AKM procedure when a new STA arrives at the AP. It consists of four phases: (A) Open Authentication and Association; (B) Mutual Authentication between the SAS and the AAS; (C) Establishing a SA between the AP and the SAS; and (D) Establishing a SA between the STA and the AP. Phase (B) and (C) are executed only once when the first STA belonging to the SAS attaches to the AP.

Table 2 specifies the notations used throughout this section and their corresponding definitions. The terms, functions, and control messages not defined in the table are sourced from the IEEE 802.11-2012 standard. The protocol specified below and presented in Figure 4 shows the newly included parameters and those parameters related to the key derivation. All other parameters used at the IEEE 802.1X-2004 and the four-way handshake at IEEE 802.11-2012 follow set standards.

### 4.1. Phase A: Open Authentication and Association

This phase follows the open authentication and association procedure defined in the IEEE 802.11-2012 standard. An IoT domain element (IDE) is included in the beacon of an AP if the AP supports the IoT association described in this section. The IDE is a newly defined information element that has an access network identifier of two octets in length. The STA selects an AP that advertises the access network identifier matching an entry in the list of potential access network identifiers with which it can associate. The STA then performs the open authentication and association procedure. The association request includes the IDE and the robust security network element (RSNE). The RSNE should contain the proposed IoT AKM mechanism as the value of the AKM suite field. After the open authentication and association procedure, the STA starts the AKM procedure by sending the EAPOL-Start message to the AP. The message includes a network identity (NID) set TLV. The NID-name field of the NID Set TLV contains the SAS-ID. The STA must remember the EAPOL-Start message that will be used later for AUTH element calculation.

### 4.2. Phase B: Mutual Authentication between the SAS and the AAS

When the AP receives the EAPOL-Start message, it looks at the SAS-ID in the message and distinguishes the SAS from the SAS-ID. It then checks whether it has established a SA with the SAS. If such a SA does not exist, the AP starts the authentication with the SAS. Otherwise, the AP skips phase B and C and starts phase D. The authentication follows the procedure described in IEEE 802.1X-2010, Section 8. The SAS performs the supplicant role in this phase. The AP and the AAS act as the authenticator and the authentication server, respectively. As a result, a MSK for the AP and the SAS is thereby generated.

Note that there is no role for the SAS in this mutual authentication phase. This allows various authentication methods to be selectable that are not impacted by the resource limitations of the STA, and their future upgrades will not affect thousands of STAs.

### 4.3. Phase C: Establishing a SA between the SAS and the AP

At this phase, the AP and the SAS establish a SA. The AP and the SAS derive a PMK and a PMKID from the MSK and then derive the PTK_SAS from the PMK. The key derivations follow the methodologies defined in IEEE 802.11-2012, Section 11, although they use the SAS-ID as the supplicant address instead of the STA-ID. They execute the four-way handshake to establish the SA. The first two messages in the four-way handshake include RSNE in order to negotiate the pairwise cryptographic parameters between the SAS and the AP. The SA is used to protect messages exchanged between the SAS and the AP at phase D.

### 4.4. Phase D: Establishing a SA between the STA and the AP

At this phase, the STA and the AP establish the SA. The exchange between the SAS and the AP derives the PTK for the STA. The AP sends the first EAPOL-KEY message to the SAS. This message includes the PMKID and the STA-ID as additional parameters. The SAS derives the PTK from the PMK on behalf of the STA, since the STA does not have the RNG and KDF. The STA-ID is used as the supplicant address in the KDF. It then encrypts the PTK with the pre-configured PSK of the STA. The SAS adds the encrypted PTK to the second EAPOL-KEY message. Next, the AP derives the PTK independently using the parameters included in the second message. The SA established at phase C protects these two messages.

The PTK is installed in the STA according to the latter three messages. During this process, the STA and the AP demonstrate (to each other) whether they have the same PTK. The AP sends the third EAPOL-KEY message to the STA. It contains the encrypted PTK, the APNonce2 used at the PTK derivation, and the MIC. The STA decrypts the encrypted PTK using its PSK and verifies the MIC with the Key Confirmation Key (KCK) derived from the decrypted PTK. If the verification is successful, the STA confirms that the AP has the corresponding PTK. The STA then sends the fourth EAPOL-KEY message to the AP. This message includes the AUTH element, containing the hash value of the EAPOL-Start message sent by the STA in Phase A. The hash value is calculated with concatenating APNonce2, STAaddr, and APaddr to the EAPOL-Start message. The AP then verifies the AUTH and MIC within the message and confirms that the STA has a valid PTK. The fifth message completes the message exchange. Note that the source and destination address fields in the MAC frame can be used as the AP-ID and the STA-ID; in this case, the MIC must protect the address fields.

Note that the SAS shall transmit the nonce and initial counter value used at the PTK encryption to the AP by putting them in the Key Nonce and Key Replay Counter fields of the second EAPOL-Key message. The AP shall pass these values intact within the third message sent to the STA so that the STA uses the nonce and initial counter for decryption.

## 5. Security Evaluation

In this section, we provide details of the security analysis of the proposed AKM mechanism. The first subsection section shows that the proposed scheme provides secure key agreement and mutual authentication. Next, the subsequent subsections introduce attacks that are prevented by the proposed scheme. For the sake of convenience, we designate each of the messages used in the proposed protocol according to a phase alphabet and a message sequence number. For example, D1 represents the first message of phase D.

It should be noted that phases A, B, and C are similar to IEEE 802.11 Open Association, IEEE 802.1X, and IEEE 802.11 four-way handshake, respectively. In particular, their security-related elements (nonces and MICs) follow those of the standards. We can expect that their security capabilities will be the same as those provided by IEEE 802.11. In phase D, communication between the AP and the SAS (D1 and D2) uses the secure channel established in phase C; the exchanges involving the AP and the SAS are therefore considered to be secure. For these reasons, we mainly focus on the D3–D5 messages that are exchanged between the AP and the STA, to analyze their protection capabilities against major attacks.

### 5.1. Mutual Authentication and Secure Key Agreement

In Phase B, a trust relationship is established between the AP and the SAS, through the mutual authentication process using IEEE 802.1X. Through D1 and D2 in phase D, the AP and the SAS derive PTKs independently. Then the PTK is delivered securely from the SAS to the STA via the AP. The possession of the same PTK at the STA and the AP can provide proof of the mutual authentication of both parties. That is verified by the MICs in D3–D4.

### 5.2. Protection from Eavesdropping

If an adversary node eavesdrops on the communication between the STA and the AP, it is able to read the contents in D3–D5 and can obtain the STA-ID, AP-ID, APNonce2, the encrypted PTK and MICs. The adversary node can also know the SAS-ID at phase A. However, even with this information, the adversary cannot generate the PTK because it does not know the APNonce1 and the MSK. It cannot obtain the PTK by decrypting the encrypted PTK unless it knows the PSK, which is never transported during the entire AKM mechanism.

### 5.3. Protection from Replay Attacks

As mentioned above in Section 3.1, the proposed mechanism assumes the same key replay protection mechanism used as that in the IEEE 802.11-2012. According to the mechanism, each EAPOL-Key messages have a Key Replay Counter field, so the STA or the AP can detect the replay attack by checking the counter value.

### 5.4. Protection from Man-in-Middle Attacks

When an adversary attempts a man-in-middle attack between the STA and the AP, it cannot replace the source and destination addresses in D3–D5 because their MICs protect them. The adversary cannot generate the MICs unless they obtain the correct PTK by decrypting the encrypted PTK in D3.

### 5.5. Minimizing the Impact of a Compromised Device

When one of the STAs is compromised by any means, the impact must be minimized. An adversary can obtain the information in the compromised STA, including the STA’s PSK and PTK. However, since each STA has its own PSK, the adversary does not know the PSKs of the other STAs. Since each PTK is encrypted by the corresponding PSK when sent, the adversary cannot deduce the PTK of another STA. Thus, apart from the compromised STA, the communications of other STAs are secure from the adversary.

Even if many STAs are compromised, the confidentiality and authenticity of AP’s communication with non-compromised STAs using the same access network will not be affected. Generally, random number generators for key generation satisfy unpredictability, unbiased, and uncorrelated conditions. Therefore, knowing the keys of several compromised STAs cannot infer the key of another STA from them.

### 5.6. Resistance of SAS to DDoS Attack

We can consider that many STAs are corrupted and they send a large number of EAPOL-start messages to the AP. In this case, the processes of phase B, C, and D may occur between the AP and the SAS. However, in our protocol, phase B, C between an AP and a SAS occurs only once, so these phases do not cause service unavailability in SAS. Only the key derivation and key encryption of phase D is then repeated, without requiring mutual authentication. This greatly mitigates SAS’s burden on DDoS attacks using EAPOL-Start messages. As will be explained in the performance evaluation section, the cost of key derivation or encryption is very small compared to mutual authentication. Therefore, SAS with powerful CPU is expected to be able to withstand DDoS attacks from tens of thousands of compromised STAs.

## 6. Related Work

Bonetto et al. [11] proposed a security protocol stack for IoT networks, which includes IoT gateways (GWs). A GW is a special IoT device with unconstrained resources and adapts the communication between the other IoT devices and the remote peer via access network infrastructures. They proposed an AKM mechanism to attach an IoT device to an access network (Figure 4 in [11]). It uses EAP, carried by Protocol for Carrying Authentication for Network Access (PANA) [12] between the IoT device and the GW. The GW and the IoT device derive a PaC-EP Master Key (PEMK) [13], and shares it with the Enforcement Point (EP). Consequently, a PEMK-based secure channel is established between the IoT device and the EP.

The Trust Extension Protocol for Authentication in Networks Oriented to Management (TEPANOM) [14] is an AKM protocol used to establish a SA between an IoT device and a deployment GW in the deployment domain. It assumes an authentication agent entity named the TEPANOM Authentication Point (TAP). A pre-defined factory shared key is used for the trust relationship between an IoT device and the TAP and is stored in the IoT device before being deployed. When the IoT device attempts to attach to a GW, the GW requests the TAP to authenticate the device on its behalf. The TAP authenticates the device and derives the session key on behalf of the device.

Unlike the proposed mechanism, they are not targeted to the 802.11ah network, and we will discuss the differences between them and the proposed mechanism in the AKM procedure. In Bonetto et al.’s scheme, each attached STA mutually authenticates itself with the AS without the help of the IoT service domain to which it belongs. This strategy has two drawbacks compared to the proposed mechanism due to the nature of the IoT network. Firstly, an AP needs to cover thousands of IoT devices. Performing the full AKM procedure for each STA is therefore burdensome for the AP. Secondly, a STA might have limited resource; the cryptographic algorithms and protocols for the AKM may therefore be unsuitable for these IoT devices. In the proposed mechanism, the AP executes the mutual authentication only once and performs the key management per STA. A STA delegates its AKM to the SAS, and it only verifies the session key with the AP.

TEPANOM assumes a pre-configured trust relationship between the TAP and the access network entities (the GWs), even though they belong to different domains. The TAP has the public-keys of the GWs. Therefore the TEPANOM protocol has no mutual authentication and the session key installation between the TAP and the GWs, corresponding to phase B and C of the proposed mechanism. In addition, in the TEPANOM protocol, the IoT device does not verify the session key sent by the GW.

## 7. Performance Analysis of the Proposed Mechanism

In this section, we present the performance analysis of the proposed mechanism focusing on the resource consumption of the IoT device. It is compared to the existing IEEE 802.11-2012 with IEEE 802.1X AKM (shortly refer to it as ‘802.11 AKM’ in this section). The 802.11 AKM in this section is assumed to use the Robust Security Network Association (RSNA) default AKM suite, CTR with CBC-MAC Protocol (CCMP) cipher suite, and the EAP with Transport Layer Security protocol (EAP-TLS) [15] with RSA [16]-based certification and key exchange.

### 7.1. Comparison of Computation Costs

Table 3 represents the computation costs of cryptographic processes in 802.11 AKM. The input sizes used are taken from the IEEE 802.11-2012 and 802.1X-2010 standards, and consumed CPU cycles were calculated with the crypto++ library benchmark [17]. About 111 megacycles are used by the 802.11 AKM. The results show that the EAP-TLS authentication process consumes almost all the computation resources. KDF uses about 38% of the computation cost of the key installation (KDF and four-way handshake) process.

Table 4 shows the computation cost of the proposed AKM. The total number of cycles of the proposed AKM is six kilocycles. The delegation of the authentication process to the SAS creates this impressive computational cost reduction. The KDF delegation also reduces the computational costs of the key installation process from 10 kilocycles to six kilocycles.

We used the ATmega128 processor [18] to show how these reductions in computational resource consumption actually affect resource-constrained devices. The processor operates with 16 MIPS and mostly single clock-cycle execution. Several IoT devices, including MICA2, use it as the main processor. As a result of dividing the CPU cycles calculated above by the computation power of ATmega128, the processor expected to use about 7 s to perform the cryptographic functions of the 802.11 AKM once, whereas it is expected to use about 0.38 ms for the proposed AKM.

### 7.2. Comparison of Network Costs

Baños-Gonzalez et al. [19] introduced an equation to obtain the time consumed for transferring a message over the IEEE 802.11ah network:

(1) $$T_{message}=DIFS+T_{DATA}+SIFS+T_{ACK}+T_{BACKOFF}+2\delta ,$$

where DIFS is the Distributed Inter-Frame Space, $T_{DATA}$ is the transmission time of the sending frame, SIFS is the Short Inter-Frame Space, $T_{ACK}$ represents the duration of acknowledgment frame transmission, $T_{BACKOFF}$ is the time to backoff, and δ is the propagation delay.

The $T_{DATA}$ in Equation (1) is attained by the Equations (2) and (3) when the normal guard interval (GI) is used. The $T_{ACK}$ calculation also employs the same equations, but the 32-byte ACK frame size is used instead of the $L_{frame}$.

(2) $$T_{DATA}=T_{Preamble\&Header}+40\times \left(N_{LTF}-1\right)+T_{sym}\times N_{sym},$$

(3) $$N_{sym}=\frac{1}{N_{DBPS}}\cdot \left\{8+6\times N_{ES}+8\times K\times L_{frame}+\left(K-1\right)\times L_{deli}\times 8\right\}$$

where $T_{Preamble\&Header}$ is the duration of the PHY preamble and header transmission, $N_{LTF}$ is the number of long training symbols, $T_{sym}$ is the duration of a symbol, $N_{ES}$ is the number of encoders, *K* is the number of aggregated frames (of equal size), $L_{frame}$ is the length of the sending frame (including header and data), $L_{deli}$ is size of the delimiter between aggregated frames, and $N_{DBPS}$ represents the number of data bits per symbol in the channel bandwidth (CBW) and the modulation and coding scheme (MCS) used for the transmission.

We use these equations to calculate the network cost of the 802.11 AKM and the proposed AKM in the IEEE 802.11ah network. The parameter values are taken from IEEE 802.11ah-2016 draft standard [20] and the referenced paper [19]. We assumed the use of the CBW as 1MHz with the MCS10. $L_{frame}$ of the 802.11 AKM messages are taken from captured messages at an IEEE 802.11 network, and their header sizes are reduced to the short header defined by the IEEE 802.11ah. Table 5 shows the parameter values used in these calculations.

Table 6 shows the calculated network costs in terms of the time consumed for the 802.11 AKM and the proposed AKM. In 802.11 AKM, the STA and the AP exchange 7259 bytes over 25 messages, consuming 472.87 milliseconds. IEEE 802.1X exchanges use about 80% of the network costs. On the other hand, the proposed AKM exchanges 1298 bytes over nine messages and consumes 100.13 milliseconds. This is about 21% of the network cost of 802.11 AKM. This reduction in network costs is due to the delegation of the IEEE 802.1X exchange. In other parts, the proposed AKM introduced additional information elements such as IDE, SAS-ID, and AUTH, which slightly increase the network cost, but they do not significantly impact the overall network cost reduction.

Considering that a single AP may associate with up to several thousand IoT devices, 802.11 AKM occupies access network resources too long per device. Therefore, the network cost reduction of the proposed scheme is effective not only for the STA resources but also for the access network conditions.

In fact, in order to calculate the total cost of the network more accurately, the network cost due to the communication between the AP and the SAS should be considered in addition to the above results. However, wired communications currently used on the Internet have a data rate of over 100 Mbps. This means that its throughput is hundreds of times larger than IEEE 802.11ah (which has 150 kbps data rate with parameter assumptions in Table 5). Thus, the time spent in wired communication is relatively very short and its cost is negligible accordingly.

### 7.3. Memory Consumption of STA

The delegation of cryptographic function of the proposed AKM also reduces memory resource consumption. The proposed AKM uses only AES in the STA, while the 802.11 AKM requires RSA and SHA1 additionally. Given the insufficient memory size of IoT devices, the difference in memory consumption due to the required cryptographic function also affects performance.

Table 7 shows the estimated memory consumption due to the cryptographic algorithms. The binary size of each of the cryptographic algorithms are obtained by compiling the axTLS library [21]. The axTLS is designed for platforms with small memory requirements, so it is suitable for measuring the minimum memory size required for implementation on memory limited IoT devices. The results show that the proposed AKM uses about 34% of the memory for cryptographic functions compared to 802.11 AKM. Compared with the 128KB program flash memory of MICA2, the proposed AKM takes up 3.9% of the flash memory and the 802.11 AKM takes 11.7%.

## 8. Conclusions and Future Work

The proposed AKM mechanism establishes an SA between a resource-constrained IoT device and an AP of an accessing network. It minimizes the number of cryptographic processes required on the IoT device by delegating the mutual authentication and the KDF processes of the device to the SAS. Compared to IEEE 802.11 with IEEE 802.1X AKM, the proposed mechanism reduces almost all the computational costs and about 79% of network costs.

In addition, cryptographic functions can be replaced with new ones without affecting the resource-constrained devices. The proposed mechanism also reduces the authentication workload of the AP as the AP authenticates the SAS only once for all STAs belonging to the SAS. This improves the scalability of the AP to a large number of STAs. Other key management protocols, such as re-keying, can be easily applied to the proposed mechanism.

For future work, we intend to study hierarchical keying for Extended Service Set (ESS). By using hierarchical keying, the SAS authenticates with an ESS, rather than with an AP, and the APs that belong to the ESS can establish SAs with the STAs that are registered at the SAS, without further authentication.

## Figures and Tables

**Table 1:** Notations for the Figure 2.

Symbol	Definitions
RSNE	Robust Security Network Element defined at IEEE 802.11
MSK	Master Session Key established via IEEE 802.1X
PMK	Pairwise Master Key derived from MSK
KDF	Key Derivation Function
PTK	Session keys shared between a STA and an AP
P	Pairwise key exchange (pairwise bit set)
ANonce	Nonce value generated by the AP
SNonce	Nonce value generated by the STA
MIC	Message Integrity Code

**Table 2:** Notations for the Figure 4.

Symbol	Definitions
IDE	IoT Domain Element
NID	Network Identity Set TLV defined at IEEE 802.1X
STA-ID	MAC address of the station
AP-ID	MAC address of the AP
SAS-ID	An identifier of a SAS
PMKID	PMK Identifier defined at IEEE 802.11
PSK	Pre-shared key between a STA and the SAS to which the STA belongs
PTK_SAS	Session keys shared between an AP and a SAS
{PTK}_PSK	A PTK encrypted by the PSK
APNonce	Nonce value generated by the AP
SASNonce	Nonce value generated by the SAS
AUTH	MIC of the EAPOL-Start message
⟶	Message sent via unsecured channel
⟹	Message sent via secured channel

**Table 3:** Computation cost of STA in IEEE 802.11 with IEEE 802.1X AKM.

Cryptographic Process		Algorithm	Input Size (Byte)	CPU Cycle
EAP-TLS	Authentication	RSA 1024 Signature	128	108.8 M
	Key exchange (supplicant side)	RSA 1024 Encryption	48	2.4 M
KDF	PMK→PTK	HMAC-SHA1x4	72	2800.4
	PMKID	HMAC-SHA1	34	1073.2
4-way HSK	Key data protection (Msg 1∼3)	AES key wrap	64	522.8
			64	522.8
			64	522.8
	MIC (Msg 2∼4)	HMAC-SHA1	143	1713.6
			143	1713.6
			99	1528.8

**Table 4:** Computation cost of STA in the proposed AKM.

Cryptographic Process	Algorithm	Input Size (Byte)	CPU Cycle
AUTH (Msg D4)	AES-CMAC	194	1102.6
Key data protection (Msg D3∼D4)	AES-CTR	130 60	1021 965
MIC (Msg D3∼D5)	AES-CMAC	229 159 99	1221.6 983.6 779.6

**Table 5:** Parameter assumption of IEEE 802.11ah message transfer delay calculation.

Parameters	Values	Note
SIFS	160 μs	At CBW 1 MHz
DIFS	264 μs	At CBW 1 MHz
$T_{BACKOFF}$	0 μs	Idle medium
δ	1.7 μs	500 m distance
$T_{Preamble\&Header}$	560 μs	At CBW 1 MHz
$N_{LTF}$	1	At single spatial stream
$T_{sym}$	40 μs	At normal guard interval
$N_{ES}$	1	Fixed for binary convolution coding
*K*	1	No frame aggregation
$L_{deli}$	4	Fixed at 11 ah
$N_{DBPS}$	6	At CBW 1 MHz with MCS10

**Table 6:** Network cost of STA in 802.11 AKM and the proposed AKM over IEEE 802.11ah network.

	Network Process Related to STA	# of msg	Sum of $L_{\mathrm{frame}}$ (Byte)	Sum of Calculated $T_{\mathrm{message}}$ (ms)
802.11 AKM	Open Association and Authentication	5	597	49.01
	IEEE 802.1X with EAP-TLS	16	6040	376.96
	4-way HSK	4	622	46.91
proposed AKM	Open Association and Authentication (A1∼A6)	6	747	60.45
	Establishing SA b/w STA and AP (D3∼D5)	3	551	39.68

**Table 7:** Memory consumption of STA due to cryptographic algorithms.

AKM Mechanism	Cryptographic Algorithm	Binary Size (Byte)	Memory Consumption (Byte)
IEEE 802.11 AKM	RSA SHA1 AES	6472 3392 5096	14,960
Proposed AKM	AES	5096	5096
